# Preterm Birth, Age at School Entry and Educational Performance

**DOI:** 10.1371/journal.pone.0076615

**Published:** 2013-10-16

**Authors:** David Odd, David Evans, Alan Emond

**Affiliations:** 1 Neonatal Unit, North Bristol NHS Trust, Bristol, United Kingdom; 2 Centre for Child and Adolescent Health, University of Bristol, Bristol, United Kingdom; University of Alabama at Birmingham, United States of America

## Abstract

**Objective:**

To investigate if the lack of gestational age correction may explain some of the school failure seen in ex-preterm infants.

**Design:**

A cohort study based on the Avon Longitudinal Study of Parents and Children (ALSPAC). The primary outcome was a low Key Stage 1 score (KS1) score at age 7 or having special educational needs (SEN). Exposure groups were defined as preterm (<37 weeks gestation, n = 722) or term (37–42 weeks, n = 11,268). Conditional regression models were derived, matching preterm to term infants on date of birth (DOB), expected date of delivery (EDD) or expected date of delivery and year of school entry. Multiple imputation was used to account for missing covariate data.

**Results:**

When matching for DOB, infants born preterm had an increased odds of a low KS1 score (OR 1.73 (1.45–2.06)) and this association persisted after adjusting for potential confounders (OR 1.57 (1.25–1.97)). The association persisted in the analysis matching for EDD (fully adjusted OR 1.53 (1.21–1.94)) but attenuated substantially after additionally restricting to those infants who entered school at the same time as the control infants (fully adjusted OR 1.25 (0.98–1.60)). A compatible reduction in the population attributable risk fraction was seen from 4.60% to 2.12%, and year of school entry appeared to modify the association between gestational age and the risk of a poor KS1 score (p = 0.029).

**Conclusions:**

This study provides evidence that the school year placement and assessment of ex-preterm infants based on their actual birthday (rather than their EDD) may increase their risk of learning difficulties with corresponding school failure.

## Introduction

It is well recognised that infants born preterm, both at extreme gestations (e.g. less than 32 weeks) but also at more modest gestations (e.g. 32–36 weeks), have worse outcomes at school age, including cognitive abilities [1], [2] and educational performance [3], [4]. While direct neurological injury is an important component of this (particularly in the very preterm group [5]) the early delivery itself may have consequences on the measurement of these age-dependent variables.

However, much of the poor long term outcome of ex-preterm infants may be exacerbated by other, modifiable factors [6]. Indeed there is some evidence that impact on educational outcomes is out of proportion to the cognitive deficits seen [1] and therefore other consequences of the preterm birth may also have a role to play.

In the UK all children are offered a school placement based on their date of birth. Consequently preterm infants may well be enrolled in school a year earlier than would be expected if this decision had been based on their expected data of birth (i.e. corrected for their prematurity). Even in the absence of neurological sequelae this may set unrealistic expectations for educational targets and leave the child constantly struggling to keep up, with potential impacts on social integration and self-esteem. Indeed nearly 30% of the most extreme preterm infants could be enrolled in school a year earlier than predicted by the expected due date.

Previous studies frequently compare outcomes between preterm infants and matched term controls, matching by date of birth, irrespective of their year of education [7]. The aim of this work is to investigate if the lack of age correction and year of education might explain some of the school failure seen in ex-preterm infants.

## Methods

The cohort in this study is drawn from the Avon Longitudinal Study of Parents and Children (ALSPAC), an on-going longitudinal study containing data on over 14,000 infants. The cohort includes children born in the Bristol area, England from April 1991 to December 1992 [8]. Further information about the study can be found on the ALSPAC website: www.alspac.bristol.ac.uk.

Data on gestational age were extracted from information routinely recorded in the clinical notes. If the gestation was recorded as less than 37 weeks (based on last menstrual period, ultrasound or paediatric assessment) then gestational age was confirmed by a single paediatrician after reviewing the clinical records.

School performance was measured using the standard UK educational measures, where progression through the school system is divided into four key stages, with examinations completed at the end of each key stage. Key stage one (KS1) comprises school years one and two (ages 5–7 years) with the KS1 test applied to all children in mainstream education at the end of this period. Children are expected to score two or above in each of the three domains (reading, writing and mathematics) and in this analysis a low score was defined as <2.

In addition, at the age of 8 years, the child’s teacher was sent an ALSPAC questionnaire, which asked the teacher to identify “Has this child ever been recognised as having special educational needs?”. The two primary outcomes were a low KS1 score or having teacher-reported special educational needs (SEN).

The following perinatal and social factors were recorded for the infants:

- Social factors: Maternal age, socioeconomic group [9] and education, car ownership, housing, crowding index (number of household members per room) and ethnicity.
- Antenatal factors: Gender, parity, weight, length and head circumference at birth.
- Intrapartum factors: Mode of delivery, maternal hypertension and pyrexia.

The dataset contained information on 13,987 infants born alive at between 23 weeks and 42 weeks of gestation. Infants were defined as preterm (less than 37 weeks, n = 885) or term (37–42 weeks, n = 13,102). A total of 1997 infants had neither of the primary outcome measures available, leaving 11,990 infants. Since not all infants had complete outcome data each analysis contained different numbers of children. Initially the differences between those infants with outcome data and those without were assessed. The characteristics of the population were assessed, split by their gestational age.

In the initial analysis, the association between gestational age group and school performance was assessed by randomly matching each preterm infant with up to 10 term infants with a date of birth (DOB) within the same calendar month. Conditional regression models (with robust standard errors) were derived using outcome and exposure measures (as binary variables) and grouping on the month of birth, while adjustment for possible confounders was performed by adding the variables described above to the models, in blocks of common variables (e.g. social factors). To minimise any potential selection bias in the multivariable models, a multiple imputation data technique (Chained Equations) was used to allow us to report on the same number of subjects for crude and adjusted analyses [10]. All variables presented in the paper (including exposure and outcome variables) were included in the imputation model although each analysis (below) was restricted to those infants with exposure (gestational age) and the appropriate outcome measure (KS1 or SEN). Further details of the imputation method are available in Table S1.

The analysis was repeated two further times. In the second analysis we matched each preterm infant with 10 term infants by their expected date of delivery (EDD) (as opposed to their actual date of birth). In the third analysis we matched by EDD and year of school attendance. The year that the infants entered school was based on the child’s date of birth (as is standard practice). This last model was weighted, using inverse probability weights, to represent the initial cohort (rather than bias it to less preterm infants). The random matching of preterm with term infants was performed independently for each cohort, and so in each the preterm infants are matched with a slightly different random selection of term infants.

Population attributable risk fractions were calculated using the odds ratio from the final adjusted model and the initial population prevalences [11].

Two sensitivity analyses were performed. In the first, the conditional regression mode was repeated, but this time using the gestational exposure group was split into three categories; very preterm (<32 weeks), moderate preterm (32–36 weeks) and term (37–42 weeks) in order to give an estimate for each of the two preterm groups. In the second, the regression model was repeated using only those infants with complete data on exposure, outcome and covariates (i.e. a complete case analysis).

All analyses were conducted with Stata 10 (Stata Corp, TX, USA). All results are presented as odds ratio (OR) (95% confidence interval (CI)), mean (SD), median (interquartile range (IQR)), or number (percent (%)).

### Ethics Statement

Written, informed consent was obtained from all mothers who entered the ALSPAC study, which was approved by the Local Research Ethics Committees. Ethical approval for this analysis was obtained from the ALSPAC Law and Ethics Committee.

## Results

### Sample

The median gestation in the preterm group was 35 (33–36) weeks, compared to 40 (39–41) weeks in the term group. Preterm infants were smaller, had lower Apgar scores and were more likely to receive resuscitation (Table 1, all comparisons p<0.001). Their intrapartum factors also differed as expected, with a higher rate of multiple births and a different profile for their mode of delivery. Mothers of preterm infants also showed different demographics from the mothers of term infants (e.g. they were younger (p = 0.04) and had less educational qualifications (p = 0.04)).

**Table 1:** Characteristics of study population (n = 11,990).

Measure	Number with data	Preterm (<37 weeks)(n = 722)	Term (37–42 weeks)(n = 11268)	P
**Pre-pregnancy factors**				
Maternal age	11,990	27.5 (4.9)	27.9 (4.9)	0.0371
Maternal socioeconomic group	9,885			0.516
I – Professional		22 (4.0%)	2,758 (29.5%)	
Ii – Managerial		163 (29.6%)	3,692 (39.6%)	
iiiN – Skilled non-manual		223 (40.6%)	1,124 (12.0%)	
iiiM – Skilled manual		76 (12.8%)	1,038 (11.1%)	
iv - Semi-skilled		52 (9.5%)	238 (2.6%)	
v – Unskilled		14 (2.6%)	485 (5.2%)	
Mother’s highest educational qualification*	10,663			0.039
CSE		155 (25.8%)	2102 (20.9%)	
Vocational		61 (10.1%)	1044 (10.4%)	
O Level		201 (33.4%)	3593 (35.7%)	
A Level		131 (21.8%)	2181 (21.7%)	
Degree		54 (9.0%)	1141 (11.3%)	
Housing	11179			0.346
Mortgaged or owned		479 (71.7%)	7804 (74.3%)	
Rented from municipality		110 (16.5%)	1564 (14.9%)	
Private Rented		79 (11.8%)	1143 (10.9%)	
Car ownership	11184	583 (87.4%)	9414 (89.5%)	0.087
Crowding index (no. people per room)	11,023			0.476
<0.5		279 (42.9%)	4202 (40.5%)	
0.5–0.75		327 (50.2%)	5455 (52.6%)	
0.75–1		29 (4.5%)	511 (4.9%)	
1+		16 (2.5%)	204 (2.0%)	
Non-white ethnicity	11828	60 (8.5%)	562 (5.1%)	<0.001
**Antenatal and intrapartum factors**				
Primiparous	11105	350 (52.2%)	5891 (56.5%)	0.037
Maternal Hypertension	10,973	25 (3.7%)	290 (2.8%)	0.205
Maternal Pyrexia	10,973	6 (0.9%)	57 (0.6%)	0.279
Multiple birth	11,990	136 (18.8%)	179 (1.6%)	<0.001
**Delivery**	10.972			<0.001
Spontaneous cephalic		400 (58.5%)	7851 (76.3%)	
Emergency caesarean section		155 (22.7%)	597 (5.8%)	
Elective caesarean section		35 (5.1%)	432 (4.2%)	
Instrumental		59 (8.6%)	1266 (12.3%)	
Breech		35 (5.1%)	142 (1.4%)	
**Infants and post-partum factors**				
Male	11,990	411 (56.9%)	5757 (51.1%)	0.002
Birth Weight (g)	111977	2356 (624)	3455 (485)	<0.001
Birth Length (cm)	10.309	47.1 (2.7)	50.9 (2.4)	<0.001
Head Circumference (cm)	10,953	32.4 (2.1)	34.9 (1.4)	<0.001
Apgar at 1 minute	10,953	7.7 (1.9)	8.4 (1.4)	<0.001
Apgar at 5 minute	10942	9.2 (1.1)	9.5 (0.7)	<0.001
Received resuscitation	10,960	173 (25.4%)	809 (7.9%)	<0.001
Died before 8 years of age	11,990	1 (0.14%)	5 (0.04%)	0.273

Standard deviations are given for means of normally distributed continuous variables and percentages for proportions.

* CSE = Certificate in Secondary Education (commonly taken at 16 years of age); Vocational = City & Guilds (intermediate level), technical, shorthand or typing, or other qualification; O level = Ordinary level (commonly taken at 16 years of age); A level = Advanced level (commonly taken at 18 years of age), state enrolled nurse, state registered nurse, City & Guilds (final or full level) or teaching qualification; Degree = University degree.

Infants not included in any analysis due to missing data on both outcomes differed from those included. In total 1997 (14.2%) infants had missing data on both outcomes. They were more likely to come from older mothers, who tended to have less educational qualifications, were less likely to own a house and more likely to be from a non-white ethnic group. Infants with missing data were more likely to have come from primiparous mothers and had lower Apgar scores at 1 and 5 minutes. They were also more likely to have received resuscitation and to have died before 8 years of age (Table S2) although they had similar gestational ages (p = 0.1367).

### Outcomes

**Figure 1:**
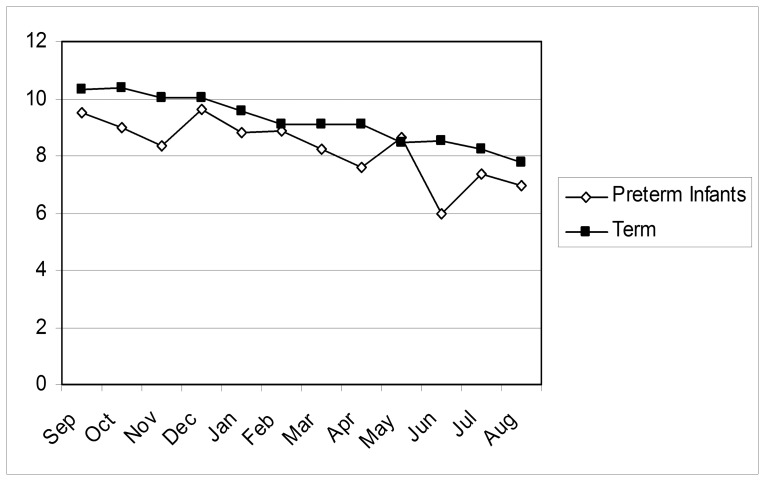
Mean Key Stage 1 scores by gestation and month of birth.

Figure 1 shows the mean summary KS1 scores by month of birth, split by gestation group (range of scores 0–15). The term infant graph shows a gradual reduction in mean KS1 scores for infants after September (and hence younger in the school year). For each month the infant was born after September the summary KS1 score dropped by a mean of 0.23 (95% CI 0.22–0.25) points (p<0.001). Preterm infants showed a similar pattern, although the lowest mean value was two months earlier in June.

Overall, preterm infants were also more likely to have a low KS1 score (31.5% vs. 21.2%, p<0.001)) and receive special educational needs support (35.5% vs. 23.3%, p<0.001) than their term peers (Table 2). Infants placed in the correct school year for their EDD had higher summary SATS scores than those in the incorrect year (9.2 (3.8) vs. 7.4 (3.6), p<0.001), and this association remained when restricting to term infants (9.3 (3.7) vs. 7.6 (3.6), p<0.001).

**Table 2:** Key Stage 1 (KS1) scores and need for Special Educational Needs support, split by gestation.

Measure	Number with data	Preterm (<37 weeks)	Term (37–42 weeks)	P
Key Stage 1 Score	11158	8.13 (4.00)	9.19 (3.74)	<0.001
Reading	11155	2.92 (1.59)	3.29 (1.50)	<0.001
Writing	11156	2.32 (1.29)	2.64 (1.24)	<0.001
Maths	11151	2.89 (1.48)	3.25 (1.37)	<0.001
Low KS1 score	11169	215 (31.5%)	2220 (21.2%)	<0.001
Receiving special educational needs support	6174	128 (35.5%)	1355 (23.3%)	<0.001

Measures are mean scores (SD), or number (%) as appropriate.

When matching for DOB, infants born preterm had an increased odds of a low KS1 score (OR 1.73 (1.45–2.06)) which persisted after adjusting for potential confounders (OR 1.57 (1.25–1.97)) (Table 3). The association persisted in the analysis matching for EDD (fully adjusted OR 1.53 (1.21–1.94)) but attenuated substantially after additionally restricting to those infants who entered school at the same time as the control infants (fully adjusted OR 1.25 (0.98–1.60)). A similar profile of attenuation existed when looking at the association between prematurity and special educational needs. When matching for DOB, infants born preterm had an increased odds of SEN (OR 1.79 (1.41–2.27)) which persisted after adjusting for potential confounders (OR 1.57 (1.19–2.07)). This association remained in the analysis matched for EDD (OR 1.59 (1.20–2.11)) but again attenuated substantially when restricting to those who entered schooling in the same year as the term infants (OR 1.13 (0.81–1.56)). There was evidence that the year of school entry modified the association between gestational age and the risk of a poor KS1 score (p_interaction_ = 0.029), although less evidence that it modified the risk of receiving special educational needs (p_interaction_ = 0.160). The population attributable risk fraction for a low KS1 score in the DOB matched analysis was 4.60%, in the EDD matched analysis was 4.43% and in the EDD and school year matched analysis was 2.12%. The population attributable risk for special educational needs showed a similar profile (DOB 4.64%, EDD 4.52%, EDD and school year 1.18%).

**Table 3:** Association between being born preterm (<37 weeks) and school performance.

Measure	Unadjusted	Adjusted for social factors*	Adjusted for social factorsand antenatal factors* †	Fully adjusted* † ‡
**Matched for DOB**				
Low KS1 score (n = 7301)	1.73 (1.45–2.06)	1.72 (1.42–2.08)	1.59 (1.26–1.99)	1.57 (1.25–1.97)
Special Educational Needs (n = 3970)	1.79 (1.41–2.27)	1.72 (1.35–2.20)	1.67 (1.28–2.18)	1.57 (1.19–2.07)
**Matched for EDD**				
Low KS1 score (n = 7338)	1.79 (1.50–2.14)	1.80 (1.48–2.19)	1.61 (1.27–2.04)	1.53 (1.21–1.94)
Special Educational Needs (n = 4041)	1.80 (1.41–2.31)	1.71 (1.31–2.22)	1.64 (1.24–2.18)	1.59 (1.20–2.11)
**Matched for EDD and same year of schooling**				
Low KS1 score (n = 6158)	1.43 (1.17–1.73)	1.40 (1.12–1.74)	1.31 (1.03–1.66)	1.25 (0.98–1.60)
Special Educational Needs (n = 3339)	1.41 (1.08–1.85)	1.33 (1.00–1.77)	1.20 (0.87–1.66)	1.13 (0.81–1.56)

* Adjusted for ethnicity, housing, crowding, and maternal education, socio-economic group, car ownership and age.

†Further adjusted for gender, parity, weight, length and head circumference at birth.

‡Further adjusted for mode of delivery, maternal hypertension and pyrexia.

Measures are OR (95% CI) for preterm infants vs. term infants.

### Sensitivity Analyses

Splitting the preterm exposure group into two sub-groups produced similar (if less precise results to the main analysis) (Tables 4 and 5). Very preterm infants had increased risk of a low KS1 score (fully adjusted OR 2.67 (1.34–5.31)) in the DOB matched analysis, which remained in the EDD matched model (fully adjusted OR 2.86 (1.35–6.04)) but again was substantially attenuated when restricting to the same year of schooling (fully adjusted OR 1.43 (0.67–4.24)). The association between very preterm infants and special educational needs was less clear, with wide confidence intervals making interpretation difficult (fully adjusted results; DOB matched: OR 1.98 (0.82–4.82), EDD matched: OR 2.36 (0.98–5.67), EDD and school year: OR 1.30 (0.41–4.16)).

**Table 4:** Association between being born very preterm (<32 weeks) and school performance.

Measure	Unadjusted	Adjusted for socialfactors*	Adjusted for social factorsand antenatal factors* †	Fully adjusted* † ‡
**Matched for DOB**				
Low KS1 score (n = 971)	3.27 (2.08–5.14)	3.01 (1.82–5.00)	2.72 (1.39–5.32)	2.67 (1.34–5.31)
Special Educational Needs (n = 506)	2.49 (1.22–5.08)	2.29 (1.03–5.10)	2.17 (0.91–5.21)	1.98 (0.82–4.82)
**Matched for EDD**				
Low KS1 score (n = 943)	3.49 (2.14–5.69)	3.59 (2.11–6.09)	3.12 (1.50–6.49)	2.86 (1.35–6.04)
Special Educational Needs (n = 513)	3.08 (1.50–6.33)	2.70 (1.18–6.18)	2.41 (0.99–5.83)	2.36 (0.98–5.67)
**Matched for EDD and same year** **of schooling**				
Low KS1 score (n = 663)	1.70 (0.93–3.09)	1.75 (0.91–3.38)	1.59 (0.76–3.31)	1.43 (0.67–3.06)
Special Educational Needs (n = 353)	1.67 (0.63–4.41)	1.43 (0.45–4.50)	1.41 (0.45–4.42)	1.30 (0.41–4.16)

* Adjusted for ethnicity, housing, crowding, and maternal education, socio-economic group, car ownership and age.

†Further adjusted for gender, parity, weight, length and head circumference at birth.

‡Further adjusted for mode of delivery, maternal hypertension and pyrexia.

Measures are OR (95% CI) for preterm infants vs. term infants.

**Table 5:** Association between being born moderate preterm (32–36 weeks) and school performance.

Measure	Unadjusted	Adjusted for social factors*	Adjusted for social factorsand antenatal factors* †	Fully adjusted* † ‡
**Matched for DOB**				
Low KS1 score (n = 6330)	1.55 (1.28–1.88)	1.56 (1.27–1.93)	1.45 (1.15–1.83)	1.44 (1.15–1.82)
Special Educational Needs (n = 3464)	1.72 (1.33–2.22)	1.66 (1.28–2.15)	1.62 (1.23–2.13)	1.53 (1.15–2.03)
**Matched for EDD**				
Low KS1 score (n = 6395)	1.60 (1.32–1.94)	1.60 (1.29–1.98)	1.45 (1.15–1.84)	1.39 (1.10–1.76)
Special Educational Needs (n = 3528)	1.68 (1.29–2.19)	1.61 (1.22–2.12)	1.56 (1.16–2.10)	1.51 (1.13–2.03)
**Matched for EDD and same year of schooling**				
Low KS1 score (n = 5495)	1.40 (1.14–1.72)	1.36 (1.08–1.71)	1.28 (0.99–1.65)	1.23 (0.96–1.59)
Special Educational Needs (n = 2986)	1.39 (1.05–1.84)	1.32 (0.98–1.78)	1.18 (0.85–1.65)	1.11 (0.80–1.55)

* Adjusted for ethnicity, housing, crowding, and maternal education, socio-economic group, car ownership and age.

†Further adjusted for gender, parity, weight, length and head circumference at birth.

‡Further adjusted for mode of delivery, maternal hypertension and pyrexia.

Measures are OR (95% CI) for preterm infants vs. term infants.

Infants born moderately preterm also showed evidence of increased risk of a low KS1 score (fully adjusted OR 1.44 (1.15–1.82)) in the DOB matched analysis, which remained in the EDD matched model (fully adjusted OR 1.39 (1.10 to −1.76)) but again was substantially attenuated when restricting to the same year of schooling (fully adjusted OR 1.23 (0.96 1.59)). A similar profile of attenuation was seen in the risk of special educational needs (fully adjusted results; DOB matched: OR 1.53 (1.15–2.03), EDD matched: OR 1.51 (1.13–2.03), EDD and school year: OR 1.11 (0.80–1.55)).

Restricting the analysis to those infants with complete data on exposure, outcomes and all covariates, provided similar (if less precise) estimates to the main analysis for a low KS1 outcome (fully adjusted results; DOB matched: OR 1.47 (1.05–2.04), EDD matched: OR 1.43 (1.01–2.02), EDD and school year: OR 1.12 (0.77–1.64)). A similar profile of attenuation existed when looking at the association between prematurity and special educational needs (fully adjusted results; DOB matched: OR 1.86 (1.23–2.80), EDD matched: OR 1.78 (1.12–2.81), EDD and school year: OR 1.30 (0.82–2.05)).

## Discussion

In this study we have shown, consistent with the existing literature, [12]–[14] that preterm infants have worse outcomes in standardised testing in primary school, and higher risk of special educational needs than their term peers. However, while these associations seemed to weaken slightly when correcting for the child’s corrected gestational age, a substantial attenuation was seen when restricting the analysis to those infants who were likely to attend school in the same year if their EDD was used (rather than their DOB). This effect was seen in infants born very preterm but also in those born between 32 and 36 weeks gestation. Indeed, there was evidence that year of schooling modifies the impact of prematurity on school outcomes, and the estimated population impact dropped once this was corrected for.

The strength of our study is the use of a population-based sample, with prospectively collected data and robust information on important confounders. The main limitation is missing data, with 14% of the eligible cohort having no outcome data, and hence selection bias from missing data is an important limitation of any interpretation of our results. However, the multiple imputation technique was used to try and reduce any impact of missing confounders and further restricting the analysis to infants with complete data produced compatible results to the main analysis. In addition there was no association between missing data and gestational age. We have also assumed that all infants entered education in the year that they were offered a place, or entered later (but into the initially offered school year). This is standard practice but it may be that some of the very preterm infants delayed their entry into school; although if this was a substantial proportion of the cohort it is likely to cause us to underestimate the true effect size. One further limitation of the ALSPAC cohort was that recruitment occurred in the 1990’s and educational processes may have changed during this time to minimise the impact of prematurity. However school failure in the ex-preterm is still very much evident in more recent work, and while general improvement may have occurred (e.g. absolute point estimates may have changed) school entry criteria have not and differential performance depending on school year is likely to remain today.

It may be that delayed school entry would benefit this cohort of infants and the results reported here are consistent with other work from the UK [4]. Some education authorities do allow individual case decisions on placing the child in an alternative year to that dictated by their date of birth (i.e. delayed school entry), or repeating a school year, although this process is not routinely offered to ex-preterm infants and may be refused [15]. However while school performance may improve, either due to better motor, attention or cognitive skill development, the social implications may not be as clear. The children will be perceived to be in a school year with children younger than them (although from the corrected-gestation perspective they are not) with corresponding impacts on social interactions with their peers. However it should be noted that the apparent reduction in educational attainment is not small. The measures of population-attributable risk suggest that around 5% of all infants failing their KS1 assessments may do so due to the impact of prematurity, although this estimate halves when we allow for their corrected age and the year of schooling.

### Conclusions

Preterm infants appear to be at higher risk of poor school performance and requiring additional educational support at primary school. Our results suggest that a proportion of the social and educational difficulties seen in these infants may be avoidable by recognising the impact that prematurity has upon school year of entry, in addition to the known impact upon cognitive and motor functions. It is possible that infants at moderate preterm gestations may similarly be affected. However whether a policy of holding infants born prematurely back to their corrected school year would have a beneficial impact is as yet unknown.

## Supporting Information

Table S1 Details of Multiple Imputation Methods.
(DOCX)

Table S2 Characteristics of infants with missing outcome data.
(DOCX)
