# Supplementary material for: Preterm Birth, Age at School Entry and Educational Performance
Source: PLoS One. 2013 Oct 16;8(10):e76615. doi: 10.1371/journal.pone.0076615 (PMC3797787; doi:10.1371/journal.pone.0076615)
Supplement: Table S1 — Details of Multiple Imputation Methods. (DOCX) [file pone.0076615.s001.docx]

Table S1. Details of Multiple Imputation Methods

All variables presented in the paper (including exposure and outcome variables) were included in the imputation model. Complete case analysis results are presented in the main text. Analysis was based on 20 imputed datasets.

| **Imputation Variable** | **n** | **%** | **Imputation Command** |
| --- | --- | --- | --- |
| Gender | 0 | 0.0% | logistic |
| Gestation | 0 | 0.0% | - |
| Multiple Birth | 0 | 0.0% | - |
| Maternal Age | 0 | 0.0% | - |
| Maternal Socioeconomic Status | 2105 | 17.6% | ordinal |
| Maternal Education | 1327 | 11.1% | ordinal |
| Parity | 885 | 7.4% | ordinal |
| Tenure | 811 | 6.8% | multinomial |
| Car Ownership | 806 | 6.7% | logistic |
| Crowding Index | 967 | 8.1% | ordinal |
| Ethnicity | 162 | 1.4% | logistic |
| Mode of delivery | 1018 | 8.5% | multinomial |
| Hypertension | 1017 | 8.5% | logistic |
| Maternal Pyrexia | 1017 | 8.5% | logistic |
| Neonatal Resuscitation | 1030 | 8.6% | logistic |
| Neonatal Sepsis | 0 | 0.0% | - |
| Birth weight | 108 | 0.9% | linear |
| Birth length | 1700 | 14.2% | linear |
| Birth head circumference | 1627 | 14.6% | linear |
| DOB | 0 | 0.0% | - |
| Incorrect schooling year | 0 | 0.0% | - |
| SEN | 5816 | 48.5% | logistic |
| Writing SATS | 834 | 7.0% | linear |
| Maths SATS | 839 | 7.0% | linear |
| Reading SATS | 835 | 7.0% | linear |
| Incorrect Year of Schooling*Preterm | 0 | 0.0% | - |
