# Supplementary material for: Preterm Birth, Age at School Entry and Educational Performance
Source: PLoS One. 2013 Oct 16;8(10):e76615. doi: 10.1371/journal.pone.0076615 (PMC3797787; doi:10.1371/journal.pone.0076615)
Supplement: Table S2 — Characteristics of infants with missing outcome data. (DOCX) [file pone.0076615.s002.docx]

**Table S2. Characteristics of infants with missing outcome data**

| **Measure** | **Number with data** | **Outcome data available**  **(n=11990)** | **Missing outcome data**  **(n=1997)** | **P** |
| --- | --- | --- | --- | --- |
| **Pre-pregnancy factors** |  |  |  |  |
| Maternal age | 13987 | 27.9 (4.9) | 28.6 (5.1%) | <0.001 |
|  |  |  |  |  |
| Maternal socioeconomic group | 11496 |  |  | <0.001 |
| I – Professional |  | 507 (5.1%) | 179 (11.1%) |  |
| Ii – Managerial |  | 2921 (29.6%) | 638 (39.6%) |  |
| iiiN – Skilled non-manual |  | 3915 (39.6%) | 490 (30.4%) |  |
| iiiM – Skilled manual |  | 1200 (12.1%) | 159 (9.9%) |  |
| iv - Semi-skilled |  | 1090 (11.0%) | 117 (7.3%) |  |
| v – Unskilled |  | 252 (2.6%) | 28 (1.7%) |  |
|  |  |  |  |  |
| Mother’s highest educational qualification* | 12404 |  |  | <0.001 |
| CSE |  | 2257 (21.2%) | 239 (13.7%) |  |
| Vocational |  | 1105 (10.4%) | 115 (6.6%) |  |
| O Level |  | 3794 (35.6%) | 512 (29.4%) |  |
| A Level |  | 2312 (21.7%) | 474 (27.2%) |  |
| Degree |  | 1195 (11.2%) | 401 (23.0%) |  |
|  |  |  |  |  |
| Housing | 13032 |  |  | <0.001 |
| Mortgaged or owned |  | 8283 (74.1%) | 1280 (69.1%) |  |
| Rented from municipality |  | 1674 (15.0%) | 187 (10.1%) |  |
| Private Rented |  | 1222 (10.9%) | 386 (20.8%) |  |
|  |  |  |  |  |
| Car ownership | 13041 | 997 (89.4%) | 1644 (88.5%) | 0.269 |
|  |  |  |  |  |
| Crowding index (no. people per room) | 12835 |  |  | <0.001 |
| < 0.5 |  | 4481 (40.7%) | 860 (47.5%) |  |
| 0.5-0.75 |  | 5782 (52.5%) | 836 (46.1%) |  |
| 0.75-1 |  | 540 (4.9%) | 75 (4.1%) |  |
| 1+ |  | 220 (2.0%) | 41 (2.3%) |  |
|  |  |  |  |  |
| Non-white ethnicity | 13648 | 622 (5.3%) | 145 (8.0%) |  |
|  |  |  |  |  |
| **Antenatal and intrapartum factors** |  |  |  |  |
| Primiparous | 12948 | 4864 (43.8%) | 875 (47.5%) | 0.003 |
| Maternal Hypertension | 12709 | 315 (2.9%) | 66 (3.8%) | 0.035 |
| Maternal Pyrexia | 12709 | 63 (0.6%) | 11 (0.6%) | 0.762 |
| Multiple birth |  |  |  |  |
|  |  |  |  |  |
| Delivery | 12705 |  |  | 0.097 |
| Spontaneous cephalic |  | 8251 (75.2%) | 1256 (72.5%) |  |
| Emergency caesarean section |  | 752 (6.9%) | 141 (8.1%) |  |
| Elective caesarean section |  | 467 (4.3%) | 72 (4.2%) |  |
| Instrumental |  | 1325 (12.1%) | 230 (13.3%) |  |
| Breech |  | 177 (1.6%) | 34 (2.0%) |  |
|  |  |  |  |  |
| **Infants and post-partum factors** |  |  |  |  |
| Male | 13985 | 6168 (51.4%) | 1072 (53.7%) | 0.058 |
| Birth Weight (g) | 13936 | 3389 (560) | 3345 (650) | 0.5701 |
| Birth Length (cm) | 11881 | 50.7 (2.6) | 50.8 (3.0) | 0.7059 |
| Head Circumference (cm) | 11990 | 34.7 (1.6) | 34.7 (1.8) | 0.649 |
| Apgar at 1 minute | 12685 | 8.3 (1.4) | 8.1 (1.7) | <0.001 |
| Apgar at 5 minute | 12668 | 9.5 (0.7) | 0.4 (1.1) | <0.001 |
| Received resuscitation | 12668 | 982 (9.0%) | 188 (10.9%) | 0.010 |
| Died before 8 years of age | 13987 | 90 (4.5%) | 6 (0.05%) | <0.001 |
| Gestation | 13987 | 40 (39-41) | 40 (48-41) | 0.1367 |

Standard deviations are given for means of normally distributed continuous variables and percentages for proportions.

* CSE=Certificate in Secondary Education (commonly taken at 16 years of age); Vocational=City & Guilds (intermediate level), technical, shorthand or typing, or other qualification; O level=Ordinary level (commonly taken at 16 years of age); A level=Advanced level (commonly taken at 18 years of age), state enrolled nurse, state registered nurse, City & Guilds (final or full level) or teaching qualification; Degree=University degree
